# Alpha-Lipoic Acid Can Overcome The Reduced Developmental Competency Induced by Alcohol Toxicity during Ovine Oocyte Maturation

**DOI:** 10.22074/cellj.2021.7071

**Published:** 2021-05-26

**Authors:** Ali Moghimi Khorasgani, Reza Moradi, Farnoosh Jafarpour, Faezeh Ghazvinizadehgan, Somayyeh Ostadhosseini, Alireza Heydarnezhad, Ali Akbar Fouladi-Nashta, Mohammad Hossein Nasr-Esfahani

**Affiliations:** 1. Department of Reproductive Biotechnology, Reproductive Biomedicine Research Center, Royan Institute for Biotechnology, ACECR, Isfahan, Iran; 2. Department of Agricultural Biotechnology, College of Agriculture, Isfahan University of Technology, Isfahan, Iran; 3. Reproduction Genes and Development Group, Department of Veterinary Basic Sciences, The Royal Veterinary College, HawksheadLane Hatfield, Herts AL97TA, UK

**Keywords:** Alcohol, Alpha-Lipoic Acid, Oocyte Maturation, Reactive Oxygen Species, Thiol

## Abstract

**Objective:**

Alpha-lipoic acid (ALA) as a strong antioxidant has a protective effect. This study was designed to assess whether supplementation of maturation medium with ALA during *in vitro* maturation (IVM) can attenuate the toxic effect of ethanol.

**Materials and Methods:**

In this experimental study, to assess the antioxidant capacity of ALA challenged by 1% ethanol during *in vitro* maturation, immature ovine oocytes were exposed to 1% alcohol in the presence or absence of 25 µM ALA during oocyte maturation. The cumulus expansion index, intracellular reactive oxygen species (ROS), and thiol content levels were assessed in matured oocytes of various treatment groups. Consequently, the blastocyst formation rate of matured oocytes in various treatment groups were assessed. In addition, total cell number (TCN), cell allocation, DNA fragmentation, and relative gene expression of interested genes were assessed in resultant blastocysts.

**Results:**

The results revealed that alcohol significantly reduced cumulus cells (CCs) expansion index and blastocyst yield and rate of apoptosis in resultant embryos. Addition of 25 µM ALA to 1% ethanol during oocyte maturation decreased ROS level and elevated Thiolcontent. Furthermore, supplementation of maturation medium with ALA attenuated the effect of 1% ethanol and significantly increased the blastocyst formation and hatching rate as compared to control and ethanol groups. In addition, the quality of blastocysts produced in ALA+ethanol was improved based on the low number of TUNEL positive cells, the increased expression level of mRNA for pluripotency, and anti-oxidant markers, and decreased expression of apoptotic genes.

**Conclusion:**

The current findings demonstrate that ALA can diminish the effect of ethanol, possibly by decreasing the ROS level and increasing Thiolcontent during oocyte maturation. Using the ALA supplement may have implications in protecting oocytes from alcohol toxicity in affected patients.

## Introduction

Ethanol can be used as a cryoprotectant (1) and also a chemical activator for artificial activation of oocytes and reconstructed oocytes (2). However, high concentrations of alcoholcan not only influence the biological nature of somatic cells but also adversely affect the germ cell ofspermatogenesis. While there are limited studies addressing the effect of ethanol on gametogenesis and preimplantation embryos (3), many studies have shown that ethanol at high concentrations can be a teratogen for developing embryos after implantation. The molecular pathway through which it induces fetal teratogenicity is well studied and has led to public awareness to avoid alcohol consumption throughout pregnancy (4).

Alcohol use and heavy drinking are common during adolescence, and its prevalence escalates into late adolescence and early adulthood, which have a devastating effect on an individual’s health (5) and may lead to abortion, birth defects, and developmental disabilities (6). In a recent study using porcine embryos, Larivière et al. (3) showed that the presence of physiological doses for several days is toxic for porcine pre-implantation embryos and leads to mitochondrial impairment. These authors attribute this effect to vulnerability of embryos during differentiation of the inner cell mass (ICM) and trophectoderm (TE) cells which requires massive reorganization at genomic, epigenomic and mitochondrial level. Similar results were reported by Maier et al. (7) who showed that alcohol toxicity at blastocyst stage cases alteration at transcriptomic level, resembling when neurons are exposed to alcohol.

Despite numerous studies on the effect of oxidative stressors on early embryos (7), to our knowledge, there are few studies thataddressed the impact of alcohol toxicity during oocyte maturation on the competency of oocytes to process the sperm genome during fertilization. Inthis regard (8), have shown that ingestion of 10% ethanol for 15 days can cause a significant reduction in the ratio of blastocyst expansion and hatching, and also impair trophoblast invasion. Furthermore (9), have revealed that treating porcine oocytes with 1 and 3% ethanol during *in vitro* maturation promotes the generation of reactive oxygen species (ROS) and diminish glutathione (GSH) level in treated oocytes.These oocytes had significantly lower cleavage rate and produced less blastocyst following *in vitro* fertilization (IVF).

Alpha-lipoic acid (ALA), as a disulfide derivative of octanoic acid, is well-known for its antioxidant capacity in various biological processes and also scavenging ROS (8). It has been proposed that ALA is a potential therapeutic agent in the treatment or prevention of different pathologies that may be related to an imbalance of oxidative cellular status (9). It has been well studied that ALA could be effective in preventing ethanol-induced neurotoxicity in the clonal hippocampal cell line HT22 (10). Furthermore, ALA inhibited toxicant-induced inflammation and ROS generation in hepatic stellate cell activation and liver fibrosis (11). In addition, many studies suggest ALA for thetreatment of diabetic peripheral neuropathy (12).

For the investigation of the possible effect of ALA to rescue the development of oocytes exposed to ethanol during oocyte maturation, this study was designed to assess whether supplementation of maturation medium with ALA during *in vitro* maturation (IVM) can attenuate the toxic effect of ethanol.

## Materials and Methods

### Media and chemicals

In this experimental study, all media and chemical reagents were obtained from Gibco (Grand Island, NY, USA) and Sigma Chemical Co. (St. Louis, MO, USA), respectively, unless otherwise specified.

All animal experiments were approved by the Institutional Review Board and Institutional Ethical Committee of the Royan Institute (95000229).

### Cumulus-oocyte complexes recovery and *in vitro* maturation 

**Fig.1:**
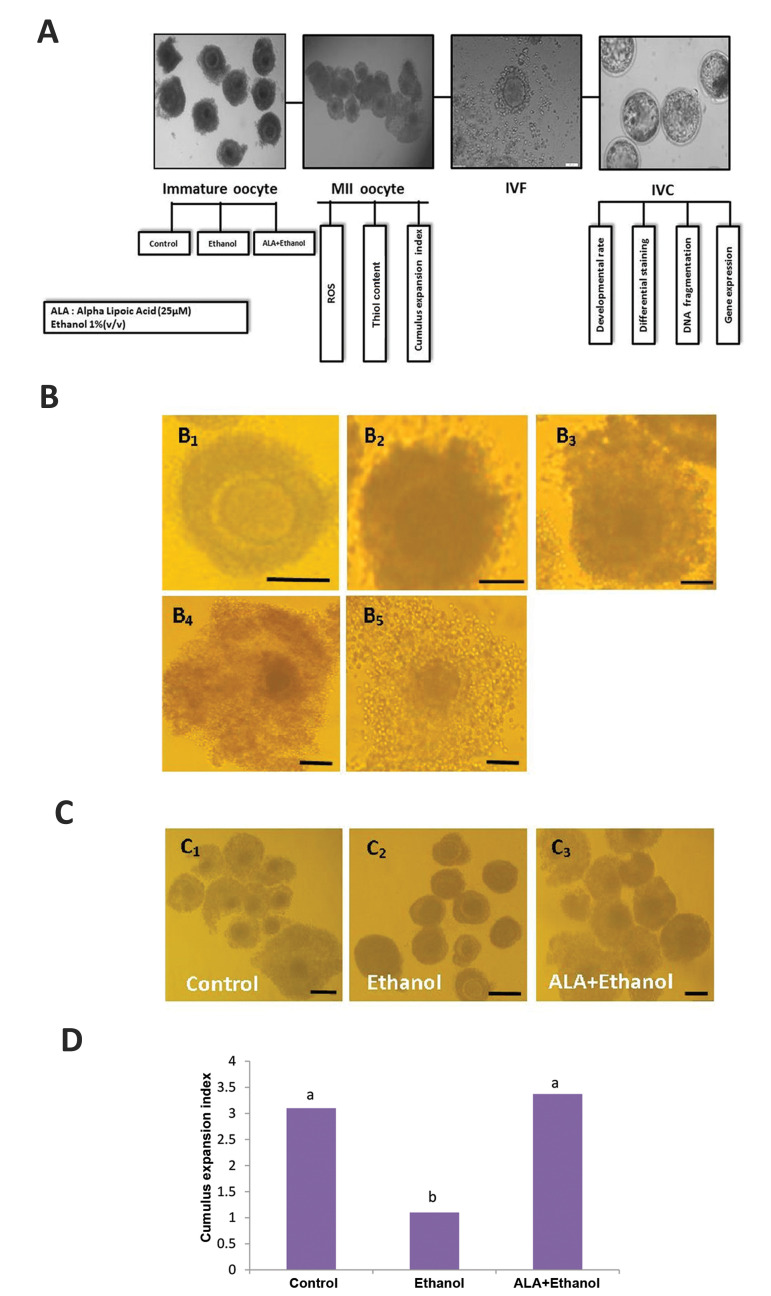
The schematic presentation of experimental design. **A.** Experimental design. According to our experiment abattoir-derived ovaries from ovine were used as the source of oocytes. Experimental groups included control, 1% ethanol and 25 µM ALA+1% ethanol groups. 22 hours after maturation of COCs in various treatment groups the cumulus expansion index was scored. Subsequently, matured COCs were stained for ROS and thiol content. Then matured COCs were transferred into droplets of fertilization medium and after the injection of sperm transferred into IVC medium. Then developmental rate, relative gene expression, DNA fragmentation, and differential staining of embryos were carried out. **B.** Morphology of COCs in different treatment groups scored 22 hours post IVM. **B_1_** **.** Score 0, no expansion, **B_2_ .** Score 1, no expansion but cells appear as spherical, **B_3_** **. **Score 2, only the outermost layers of cumulus cells have expanded, **B_4_ . **Score 3, all cell layers have expanded except the corona radiate, and **B_5_ . **Score 4, expansion has occurred in all cell layers including the corona radiate. Morphology of expansion in **C_1_ .** Control,**C_2_ .** Ethanol and **C_3_ .**ALA+ethanol groups. **D. **Expansion index of COCs in various treatment groups. Columns with different letters are considered as significant (P<0.05) (scale bars represent 200 µm). COCs; Cumulus oocyte complexes, ALA; Alpha-lipoic acid, IVC; *In vitro* culture, IVF; *In vitro* fertilization, IVM; *In vitro* maturation, and ROS; Reactive oxygen species.

Abattoir-derived ovaries from ovine were used as the source of oocytes. Ovaries were transported to the laboratory within the minimum possible time (2-3 hours) in saline solution (0.90% w/v NaCl) at 15-20˚C. After trimming and washing, they were stored for 12 hours at 15˚C (13). Cumulus-oocyte complexes (COCs) were aspirated from the antral follicle (2-6 mm diameter) with the aid of a 20-G needle attached to a vacuum pump (80 mm Hg). Thereafter, the best quality COCs with at least threelayers of cumulus cells (CCs) and, intact and evenly granulated cytoplasm were randomly allocated into one of three experimental groups (Fig .1A). COCs were matured in tissue culture medium 199 (TCM199) containing 10% fetal bovine serum (FBS), follicle-stimulating hormone(FSH, 10 µg/mL), luteinizing hormone(LH, 10 µg/mL), estradiol-17b (1 µg/mL), cysteamine (0.1 mM) (maturation medium: MM) at 38.8˚C, 5% CO_2_ and humidified air for 22 hours (14).

### Cumulus expansion index

Cumulus expansion index of COCs was scored 24 hours after maturation based on Vanderhyden et al. (15). Expansion was scored 0-4: Score 0: no expansion in CCs (Fig .1B_1_); score 1: no expansion in CCs but cells appear as spherical (Fig .1B_2_); score 2: only the outermost layers of CCs expanded (Fig .1B_3_); score 3: all layers of cells expanded except the corona radiate (Fig .1B_4_); and score 4: expansion occurred in all layers of cell (Fig .1B_5_).This experiment was done in triplicate, and in each replicate, at least 30 matured COCs were assessed.

### Measurement of thiol content

Cell Tracker™ Blue CMF2HC (4-chloromethyl-6, 8-difluoro-7-hydroxycoumarin) (C12881, Molecular Probes), a membrane-permeable fluorescence probe was used as a sensitive and specific probe to evaluate intracellular thiol content, especially GSH (16-18). Following the maturation of COCs in various treatment groups, matured COCs were denuded by vortexing for 3-5 minutes in HEPES-buffered TCM199 (H-TCM199) supplemented with 300 IU/ml hyaluronidase. Subsequently, denuded matured oocytes were exposed to 20 µM Cell Tracker Blue CMF2HC for 20 minutes at 38.5˚Cin the dark and then washed three times with phosphate buffer solution without calcium and magnesium (PBS) containing 1 mg/ml polyvinyl alcohol (PVA). The oocytes were then placed into 10 µl droplets of PBS+PVA and observed using an inverted fluorescent microscope (Olympus, IX71, Japan). Immediately after exposure, a digital image of each matured oocyte was taken with a highly sensitive camera (DP-72, Olympus, Japan) operated on DP2-BSW software. The fluorescence intensity of oocytes was analyzed using ImageJ software (National Institutes of Health, Bethesda, MD). Assessing oocyte thiol content was done in three replications, and at least 30 matured COCs were used in each replication.

### Measurement of reactive oxygen species 

The procedure for ROS measurement was as described previously (16, 17). In brief, after the preparation of matured oocytes similar to the previous section (the measurement of thiol content), oocytes were exposed to 10 µM DCHFDA (2, 7-dichloro dihydroflouresceindiacetate, Sigma, D6883) for 30 minutes at 38.5˚C in the dark and then washed extensively in PBS. For themeasurement of ROS levels, matured oocytes were exposed to UV light of a fluorescent microscope (Olympus, IX71, Japan) and observed using filter sets (excitation wavelength: 450-490 nm, emission wavelength: 515-565 nm). Taking digital images and quantification of fluorescent intensity was exactly the same as the previous section. The measurement of ROS level was done in three replications and using at least 30 matured COCs in each replicate.

### *In vitro* fertilization 

Fresh ram semen was washed and centrifuged two times and resulted pellet was re-suspended with fertilization medium containing 2 mg/ml BSA (18, 19). Matured COCs from various treatment groups were washed separately in fertilization medium, and groups of 10 matured COCs were transferred into 50 μl droplets of fertilization medium containing 2×10^6^ /ml motile sperm under mineral oil as previously described (20). The inseminated COCs were incubated for 20 hours in 5% CO_2_ in humidified air at 38.5˚C. Thereafter, presumptive zygotes mechanically denuded via pipetting and then cultured in groups of five to seven in modified synthetic oviductal fluid (mSOF) (21) under mineral oil at 38.5˚C, 5% CO_2_ , 5% O_2_ and humidified air for seven days in 20 μl droplets. The cleavage, blastocyst, and hatching rates were evaluated on days 3, 7, and 8 post-fertilization, respectively. The number of replications and matured oocytes in each group is depicted in Table 1.

### Differential staining

In order to determine the number of ICM and TE, differential staining was carried out as described previously (22). In brief, hatched blastocysts onday 8 from various treatment groups were used for staining. Thereafter, blastocysts were washed in PBS+PVA and permeabilized with 0.5% triton-X-100 in H-TCM199 containing 5 mg/ml BSA for 30 seconds. Then, blastocysts were stained with 30 µg/ml propidium iodide (PI) for 10 seconds. Subsequently, blastocysts were transferred to 10 mg/ml Hoechst at 4˚C for 15 minutes. Finally, blastocysts were mounted in mounting fluid and observed under a fluorescence microscope. ICM and TE were recognized based on their blue and red colors, respectively. Finally, the total cell number (TCN: ICM+TE) was also assessed. Totally 30 blastocysts were used for differential staining in at least three replications.

### DNA fragmentation

To determine the apoptotic cells in blastocysts from various treatment groups, in situ cell death detection kit (Promega Diagnostic Corporation, Germany), known as TUNEL (TdT-mediated dUTP-digoxigenin nick end labeling) (20). Initially, hatched blastocysts onday 8 were fixed with 4% freshly prepared paraformaldehyde for 60 minutesatroom temperature (RT). After washing the blastocysts with PBS+PVA, they were permeabilized with 0.5% triton-X-100 for 30 minutes in RT. Subsequently, blastocysts were equilibrated in equilibration buffer (EQ) in RT for 10 minutes and after that incubated in rTdT Incubation Buffer (EQ 45 µl+5 µl nucleotide mix+1 µl rTdT enzyme) for 60 minutes in 37˚C in darkness and humid environment. Immediately the reaction was inhibited by incubating the blastocysts in 2X SSC buffer for 15 minutes in RT. Finally, the blastocysts were counterstained with 10 µg/ml for 5 minutes, and after washing, they were mounted on microscopic slides and observed under a fluorescence microscope (Olympus, Japan). Total nuclei were counted by PI, and cells were considered as TUNEL positive if their nuclei showed light green. Totally 30 blastocysts were used for TUNEL assay in at least three replications.

### Gene expression analysis

Pools of expanded blastocysts in day 7 (5 in each pool) in three independent replicates were used for RNA extraction using the RNeasy Micro Kit (QIAGEN, Cat. No.74004, Germany). Reverse transcription was immediately performed using a QµantiTect Reverse Transcription (RT) Kit (QIAGEN, Cat. No.205311, Germany). The cDNA was stored at -70˚C until analysis by quantitative polymerase chain reaction (qPCR) using standard conditions. Ct values used for calculating relative expression were normalized against the reference gene (*β-ACTIN*). Three technical replicates were done in each PCR reaction that wasrepeated three times. ΔΔCT method was used to estimate fold changes between genes of interest. The primer sequences, annealing temperature, and product size are listed in Table 2.

**Table 1:** Development of preimplantation ovine embryos after treatment of immature COCs with 1% ethanol or 25 µM ALA+1% ethanol compared to the control group


Treatment	Number of oocyte	Number of cleavage (%)	Number of blastocyst (%)	Number of hatching

Control	1106	982 (86.9 ± 2.57)^a^	430 (34.19 ± 4.67)^b^	107 (27.77 ± 3.84)^a^
Ethanol	1415	1091 (75.47 ± 2.37)^b^	194 (18.19 ± 2.81)^c^	47 (16.66 ± 4.85)^b^
ALA+ethanol	1621	1522 (98.65 ± 0.37)^a^	651 (49.76 ± 1.98)^a^	136 (33.33 ± 3.61)^a^


Data are presented as mean ± SEM. Different letters in each column indicates statistically significant differences (P<0.05). COCs; Cumulus oocyte complexes and ALA; Alpha-lipoic acid.

**Table 2:** Primer sequence


Gene name	Primer sequences (5ˊ-3ˊ)	Annealing temp. (˚C)	Accession number

*β- ACTIN*	F: CCATCGGCAATGAGCGGT	58	NM_001009784.2
	R: CGTGTTGGCGTAGAGGTC		
*BCL2*	F: AGCATCACGGAGGAGGTAGAC	62	XM_012103831.2
	R: CTGGATGAGGGGGTGTCTTC		
*BAX*	F: AGCGAGTGTTCTGAAGCG	60	XM_015100639.1
	R: CCCAGTTGAAGTTGCCGT		
*CASPASE3*	F: GCTACAAGGTCCGTTATGCC	59	XM_015104559.1
	R: GATGCTGCCGTATTCGTTCTC		
*GPX4*	F: TCAATCACTTCCTCACTCAGACTG	57	XM_015096017.1
	R: GTGTGCTGGGCGACTGTATC		
*SOD1*	F: TGGCAGAGATGATACAGAGG	55	NM_001145185.1
	R: GAACTACAGCGGAGGTAAAC		
*OCT4*	F: AGCGAGTGTTCTGAAGCG	50	XM_004018968.3
	R: CCCAGTTGAAGTTGCCGT		
*NANOG*	F: ATCACCATCTTCCAGGAGCGA	54	XM_004006901.3
	R: TTCTCCATGGTGGTGAAGACG		


### Experimental design

As is demonstrated in Figure 1A, the experimental groups included: i. Control group; COCs were cultured in MM, ii. ALA+ethanol group; COCs were cultured in MM in the presence of 25 µM ALA (23) which was diluted in ethanol and final concentration of ethanol in MM reached to 1% (v/v) (24). The concentration of ALA was chosen based on the literature in farm animal species (21), iii. Ethanol group COCs were cultured in MM in the presence of 1% ethanol (v/v) based on the concentration of Ethanol which was used for dilution of ALA. 1% alcohol was used based on the legal limit of intoxication in human serum or 0.8% in the blood as the concentration of alcohol in human serum (25). In addition, we should mention that while the solvent of ALA is ethanol, we can’t have a separate ALA group.

### Data analysis

Wherever possible, data were presented as mean ± SEM. All percentage data were analyzed by SPSS16.0 statistical software (IBM Corporation, Somers, NY, USA). The normality of data and equality of variances were checked using Kolmogorov-Smirnov and Levene tests, respectively. Cumulus expansion index (CEI) was analyzed using a nonparametric Kruskal-Wallis test. Furthermore, because CEI is a nonparametric data, they were presented as only mean without any SEM. Other data were analyzed using a one-way ANOVA followed by LSD test. The differences were considered significant at P<0.05.

## Results

The effect of ethanol and alpha-lipoic acid on cumulus expansion was investigated in matured oocytes. As shown in Figure1C, D, the analysis of CEI data revealed a significant reduction in the expansion of CCs in ethanol group (1.1) as compared to control (3.1) and ALA+ethanol (3.37) (P<0.05). The CEI was similar between control and ALA+ethanol group (P>0.05).

**Fig.2:**
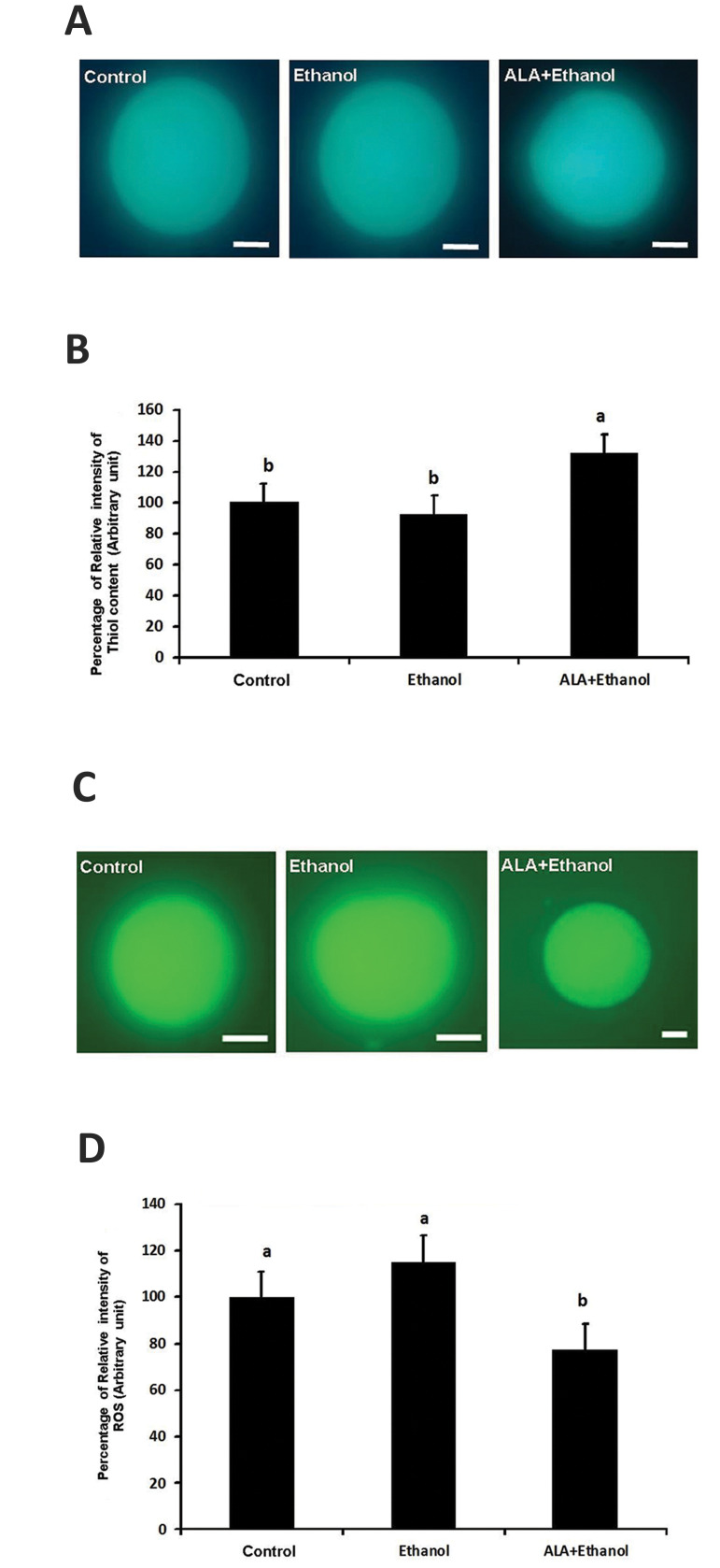
The effect of ethanol and ALA on relative ROS and thiol content of matured ovine oocytes. **A. **Representative fluorescence images of MII-oocytes for thiol content in different treatment groups groups, **B.** Percentage of relative intensity of thiol content in different treatment groups. Columns with different letters are considered as significant (P<0.05). **C.** Representative fluorescence images of MII-oocytes for ROS in different treatment groups, and **D.** Percentage of relative intensity of ROS level in different treatment groups. Columns with different letters are considered as significant (P<0.05, scale bars represent 50 µm). ALA; Alpha-lipoic acid and ROS; Reactive oxygen species.

Following staining with Cell Tracker Blue CMF2HC to assess thiol content in matured oocytes, the CMF2HC intensity in ALA+ethanol group (129.1 ± 2.11) was significantly higher than control (100) and ethanol (98.2 ± 1.54) groups (P<0.05, Fig .2A, B).

The level of intracellular ROS was assessed following staining with DCHFDA by measuring fluorescent intensity in matured oocytes. As it is depicted in Figure 2C and 2D, treatment of COCs with 25 µM ALA in the presence of 1% ethanol decreased the DCHFDA intensity (76.4 ± 1.47) as compared to ethanol (118.1 ± 1.78) and control group (100 ± 2.21) groups (P<0.05). However, there was no significant difference between control and ethanol groups (P>0.05).

In order to assess if ethanol and its combination with 25 µM ALA have any effect on the developmental competence of matured oocytes in terms of cleavage and blastocyst rates, IVF was carried out for various treatment groups. As depicted in Table 1, exposure to 1% ethanol during maturation significantly decreased the cleavage rate (75.47 ± 2.37) compared to control (86.9 ± 2.57) and ALA+ethanol group (98.65 ± 0.37, P<0.05). Furthermore, the addition of 25 µM ALA attenuated the effect of 1% ethanol and significantly increased blastocyst formation in ALA+ethanol group (49.76 ± 1.98) as compared to control (34.19 ± 4.67) and ethanol groups (18.19 ± 2.81, P<0.05). In addition, the blastocyst rate was significantly lower in the ethanol group compared to the control group (P<0.05). Finally, blastocysts hatching rate was significantly lower in the ethanol group (16.66 ± 4.85) compared to control (27.77 ± 3.84) and ALA+ethanol group (33.33 ± 3.61, P<0.05, Table 1).

**Fig.3:**
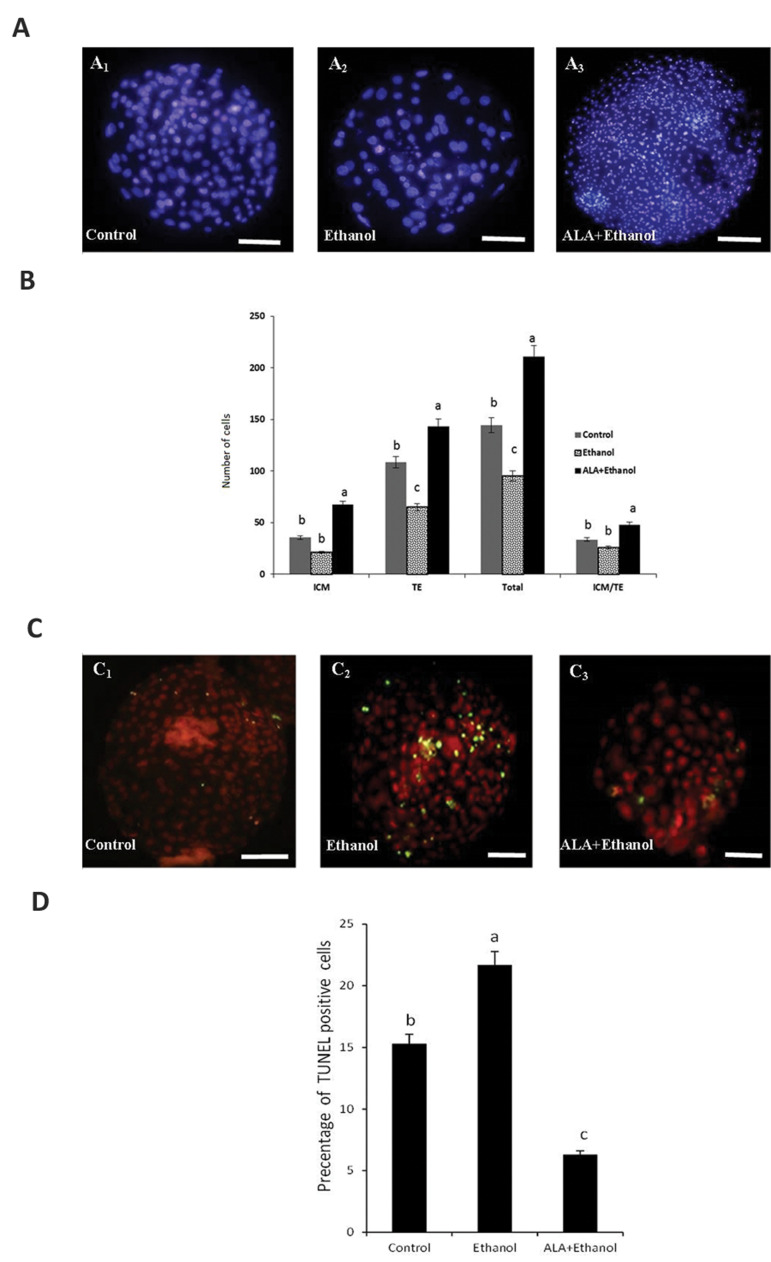
Cell number, trophectoderm and inner cell mass allocation and DNA fragmentation of cultured ovine blastocysts. **A, B.** Quality of ovine expanded blastocysts in terms of total cell number or allocation in different treatment groups (scale bars represent 100 µm). **C, D.** Quality of ovine expanded blastocysts in terms of DNA fragmentation assessed by TUNEL kit in different treatment groups (scale bars represent 50 µm). Columns with different letters are considered as significant (P<0.05).

In order to assess the quality of the blastocysts from various treatment groups, differential staining was done to determine ICM, TE, TCN, and ICM:TE ratio. As it is presented in Figure 3A, the number of TE and TCN were significantly higher in ALA+ethanol group as compared to control and ethanol group (P<0.05). In addition, the number of TE and TCNsweresignificantly lower in the ethanol group compared to the control group (P<0.05). Besides, the quality of hatched blastocysts in terms of ICM and ICM:TE was similar between control and ethanol groups (P>0.05). However, hatched blastocysts from ALA+ethanol group had significantly higher ICM and ICM:TE as compared to other groups (P<0.05, Fig .3A).

Furthermore, the effect of ethanol in the presence or absence of ALA was investigated on DNA fragmentation by TUNEL assay. As depicted in Figure 3B, the number of tunnel positive cells in the ethanol group (21.7 ± 2.41) was significantly higher than control (15.3 ± 2.12) and ALA+ethanol (6.4 ± 1.54) groups (P<0.05).

**Fig.4:**
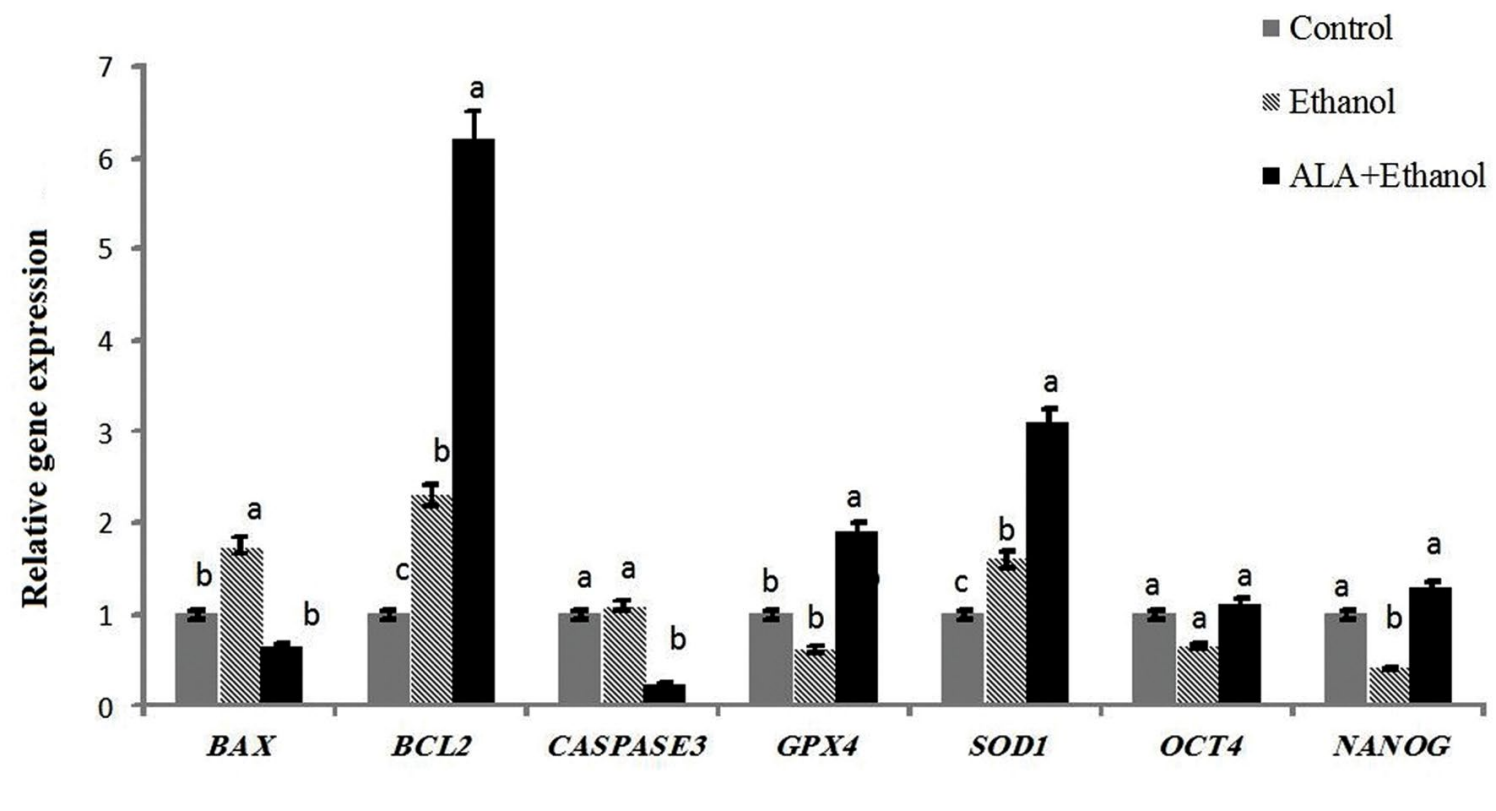
Relative gene expression of interested genes. Quality of ovine expanded blastocysts in terms of expression of genes related to apoptosis, antioxidant capacity and pluripotency in different treatment groups. Columns with different letters are considered as significant (P<0.05).

Finally, the quality of derived blastocysts was assessed in terms of expression of genes thatare related to the apoptosis pathway, antioxidant capacity, and pluripotency factors. As demonstrated in Figure 4, the expression of *BAX* was significantly lower in theALA+ethanol group compared to the ethanol group (P<0.05). However, the expression of this gene was significantly lower in the control group than the ethanol group (P<0.05). The expression of *BCL-2* as an anti-apoptotic factor was significantly higher in ALA+ethanol compared to control and ethanol groups (P<0.05).

The next gene which was assessed was *CASPASE3*, which showed significantly lower expression in ALA+ethanol COCs as compared to control and ethanol groups (P<0.05).

The anti-oxidant capacity of blastocysts derived in various treatment groups was assessed in terms of expression of *GPX4* and *SOD1*. Expression of both *GPX4* and *SOD1*was significantly higher in ALA+ethanol group as compared to control and ethanol groups (P<0.05). The expression of *OCT4* and *NANOG* was lower in the ethanol group in comparison to the control group, which was reached to a significant level for *NANOG* (P<0.05) but not for *OCT4*. However, the expression of these two pluripotency markers in theALA+ethanol group was similar to the control group.

## Discussion

Our results revealed that exposure to 1% ethanol significantly reduced the cumulus expansion and blastocyst yield. The quality of blastocysts was also reduced based on the assessment of ICM, TE, and total cell counts.

Although there are many studies in rodents that have demonstrated the effect of alcohol ingestion on sperm parameters and IVF results, there are very limited studies regarding the effect of alcohol abuse on oocytes and embryos (26). Regarding the effect of alcohol on the male reproductive system, it has been shown that alcohol drinking can alter spermatogenesis and induce morphological changes in spermatozoon (26), which may be due to alteration of the endocrine system in the male reproductive system (27).

Therefore, to assess this effect, ovine COCs were exposed to 1% alcohol during the period of maturation. To further enhance our knowledge regarding the alcohol toxicity, we assessed the degree of ROS production and GSH-antioxidant capacity of alcohol-treated oocytes. Interestingly, we found that the values of ROS and thiol content were not significantly different from control, indicating the production of ROS or reduction of antioxidant capacity does not account for the observed alcohol toxicity during oocyte maturation. One explanation for the unaltered level of thiol content may be related to the specificity of CMF2HC dye, which cannot detect other oxidized thiols and GSSG (18).

To evaluate whether apoptosis has been executed in the blastocysts derived from COCs exposed to alcohol, DNA fragmentation as the late apoptotic marker was assessed, and the results revealed a higher percentage of TUNEL positive cells in ethanol group compared to control group. Analysis of expression of *BAX, BCL2*, and *CASPASE3* as other apoptotic markers (28) and,*OCT4* and *NANOG* as pluripotent markers (29) were also assessed in the blastocyst derived from COCs treated with alcohol. Unlike *caspase3*, both *BAX* and *BCL2* were significantly altered. As expected, based on the TUNEL result, the expression of *BAX*, as a pro-apoptotic marker, was significantly higher in the alcohol group, further verifying the higher degree of apoptosis in this group. In contrary to our expectation, the expression of *BCL2*, as an anti-apoptotic marker, was also higher in the alcohol group. These data suggest that embryos thathave been able to reach blastocyst may express a higher degree of *BCL2* to overcome alcohol intoxication. The situation might have been completely different in embryos that were not competent to reach this stage, and therefore, inevitably, they were not included in our assessment.

Assessment of expression of genes related to antioxidant capacity, GPX4, and SOD1 (30, 31) revealed significantly higher expression of both SOD1 and GPX4 in the ALA+ethanol group. An increase in the expression of SOD may be related to a higher capacity of blastocysts to convert superoxide to less toxic ROS, the H_2_ O_2_ . Furthermore, higher expression of GPX4 in ALA+ethanol treated group may lead to a higher reduction of hydroperoxide groups on phospholipids, lipoproteins, and cholesteryl esters (32). In summary, the ethanol-exposed group treated with ALA demonstrated a higher anti-oxidant capacity in derived blastocysts.

In the next experiment, we evaluated how antioxidants, such as ALA, can overcome the toxic effects of alcohol. In our results, ALA overcomes the inhibitory effects of ethanol on the cumulus expansion index. It was also interesting to note that ALA improved the GSH-antioxidant capacity of the *in vitro* matured oocyte (33) and concomitantly reduced the ROS level of ALA+ethanol-treated oocytes compared to both control and ethanol groups. Interestingly, both blastocyst yield and quality of blastocysts were improved which was further verified upon assessment of TUNEL assay, mRNA expression of apoptotic markers (27, 28), pluripotent markers and anti-oxidant markers These results indicate that ALA not only can detoxify the toxicity effects of alcohol but on its own has beneficial effect on the quality of *in vitro* matured oocytes. This observation is consistent with a previous report indicating ALA+ethanol can improve the quality of oocyte during maturation (26, 27). These observed effects can be attributed to several characteristics of ALA, which are rarely observed for other antioxidants, including (33): i. Small size, ii. Both water and fat-soluble nature with rapid absorption rate, iii. Metal chelating ability, iv. ROS scavenging activity, v. Rescuing or recycling the antioxidant capacity of vitamin E and C, vi. Improving intracellular GSH level, vii. Modulator of several signaling transduction pathways like suppressing tumor necrosis factor (TNF)-alpha-induced ROS generation,and 6-hydroxydopamine induced ROSgeneration, acting as co-activator in electron chain reaction in mitochondria and thereby increasing ATP production (28) and reducing electron leakage, and viii. Acting as a coenzyme of pyruvatedehydrogenase complex and improving consumption of pyruvate especially in ovarian folliclesand in oocytes (22).

It has been shown that ALA can inhibit oxidative stress induced by arsenic or thinner and improve the quantity and quality of sperms in rats (34). In a clinical trial, the effects of ALA supplement on the spermatogram and seminal oxidative stress in infertile men wereinvestigated, and it has been revealed that total sperm count, sperm concentration, and motility levels were significantly increased in the ALA group compared with baseline values. In addition, ALA supplementation improved total antioxidant capacity compared with the placebo group (28, 34).

Our results in this study areconsistent with the previous report of Zhang et al. (22) which showed that addition of ALA at 25 μM during maturation protects oocytes from manipulation and chemical stressor and results in the improved blastocyst and a significant increase in oocyte GSH level and reduction in apoptosis rate in blastocysts (24). The positive effect of ALA also has been shown in thesomatic cell nuclear transfer (SCNT) procedure,and researchers (22, 31) have shown that the efficiency of ALA to improve SCNT in porcine is 400 times more than vitamin C.

In the nervous system (35) and blastocyst (36), ethanol activates ROS production, and protection is acquired by activation of TGFß1 and P53 pathways. Indeed, TGFß1 limits the long term damages in the brain induced by alcohol toxicity, and *in vitro*, it improves embryos quality (36). This effect is believed to be mediated by overexpression of clusterin, which is observed both in the brain and the blastocyst exposed. In this study, we did not observe the overproduction of ROS following alcohol treatment; therefore it is likely that alcohol-induced cytotoxicity during IVM is mediated through other pathways, which requires further research.Despite this, ALA may partially alleviate the alcohol toxicity, through its antioxidant nature as observed by reduced ROS and improved thiol content. Improved developmental competency following ALA treatment during IVM may be related to other properties of ALA, like modulation ofsome signaling transduction pathways such as lowering inflammation reactions (e.g., NF-KB) (37) and increasing the endogenous cellular antioxidants (e.g., GSH) (22, 38), which needs future studies.

## Conclusion

Taken together, our results in this study suggest that ALA not only overcomes the negative effect of alcohol toxicity during oocyte maturation but also improves blastocyst yield and quality of resultant embryos. Therefore, ALA, as a good supplement, or as a chemical highly available in green vegetables, is recommended for lowering ROS level and increasing the endogenous cellular antioxidants under oxidative stress conditions in the fertilization process.
